# An unusual non‐hematopoietic bone marrow finding

**DOI:** 10.1002/ccr3.8305

**Published:** 2023-12-13

**Authors:** Rahaf Altahan, Mohammed Lafi Alanazi, Mohammed Abdulaziz Alharbi, Salman Almalki, Aziza Alswayyed, Laila Alsuhaibani

**Affiliations:** ^1^ Pathology and Clinical Laboratory Medicine Administration King Fahad Medical City Riyadh Saudi Arabia

**Keywords:** artifact, bone marrow, bone marrow pathology, malignancy, sweat gland

## Abstract

We present an interesting case that showed a non‐hematopoietic structure embedded in the bone marrow biopsy. Given the clinical and morphological difficulties, it was challenging to identify this artifact's nature. Publishing this case would familiarize pathologists with this artifact and save additional testing and delays in reporting.

## Graphical abstract

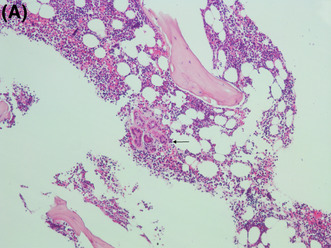

## 1 CASE

**FIGURE 1:**
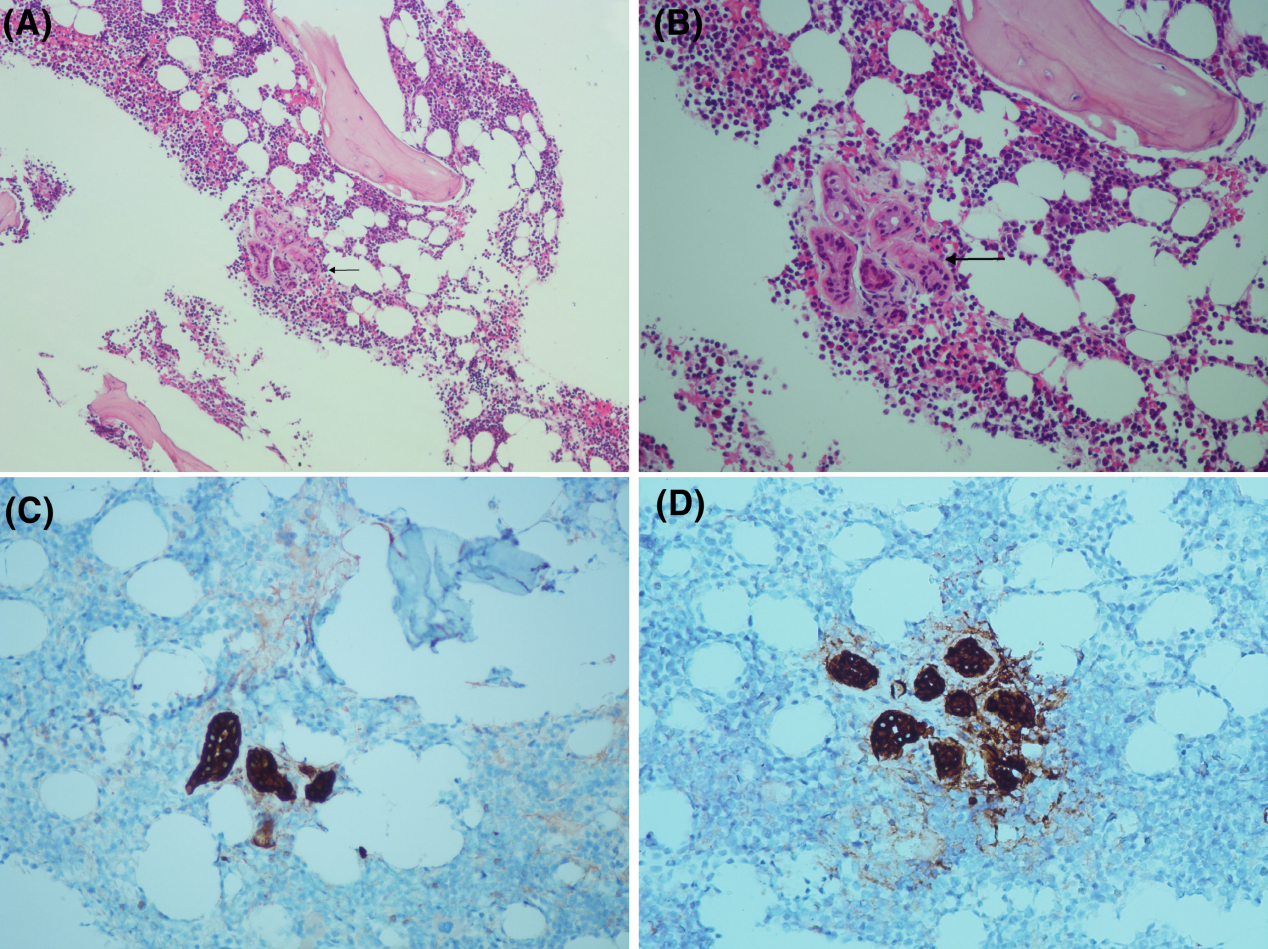
(A and B) showing H&E sections of the bone marrow trephine with the focal collection (black arrow). Note that the collection is seen between two bone marrow trabeculae and surrounded by hematopoietic elements. The focal collection showing positivity for cytokeratin 7 (C) and cytokeratin AE1/AE3 (D).

A 4‐year‐old girl presented to the emergency department with high‐grade fever, bone pain, weight loss, and multiple skin nodules. Symptoms started to develop after she was diagnosed with hand‐foot‐mouth disease 5 months before this admission, followed by prolonged fever and myalgia. The initial workup showed mild leukopenia and a positive antinuclear antibody. Examination and initial imaging revealed prominent bilateral cervical lymph nodes and splenomegaly. A bone marrow biopsy was done for assessment before starting a course of steroids; it showed a cellular and reactive bone marrow with trilineage hematopoiesis and a single well‐defined focal collection, embedded within the bone marrow area (Figure 1A,B). Some immunohistochemical stains (IHC) were requested to identify this collection's nature, including cytokeratin 7 and cytokeratin AE1/AE3 (demonstrated in Figure 1C,D). IHC enabled the identification of the collection as an eccentric sweat gland that was introduced to the bone marrow area during the bone marrow collection procedure.^1^, ^2^ With a normal bone marrow result, an MRI was further done that showed extensive myositis. As a result of the imaging and cutaneous manifestation, the patient was diagnosed with juvenile dermatomyositis and was started on steroids and methotrexate. The case was clinically and histologically challenging, as the structure was located within the bone marrow area, between trabeculae, as depicted in Figure 1A,B, and at the same cut level as the trephine biopsy. Correct identification of such artifactual lesions is imperative to avoid misdiagnosing a non‐hematopoietic involvement, especially in challenging cases such as the presented one.^3^

## AUTHOR CONTRIBUTIONS

**Rahaf Altahan:** Investigation; project administration; visualization; writing – original draft; writing – review and editing. **Mohammed Lafi Alanazi:** Writing – review and editing. **Mohammed Abdulaziz Alharbi:** Writing – review and editing. **Salman Almalki:** Investigation; methodology; visualization; writing – original draft; writing – review and editing. **Aziza AlSwayyed:** Investigation. **laila alsuhaibani:** Investigation.

## CONFLICT OF INTEREST STATEMENT

The authors have no conflicts of interest to declare.

## CONSENT

Written informed consent was obtained from the patient's parent to publish this report in accordance with the journal's patient consent policy.

## Data Availability

Data available on request from the authors.
